# From Personal Statement to Mission Statement

**DOI:** 10.7759/cureus.19426

**Published:** 2021-11-10

**Authors:** Paul Bernstein, Benjamin Doolittle

**Affiliations:** 1 Internal Medicine, Yale School of Medicine, New Haven, USA; 2 Internal Medicine-Pediatrics, Yale School of Medicine, New Haven, USA

**Keywords:** eras protocol, residency interview, personal statement, graduate medical education (gme), residency application process

## Abstract

In recent years, the application process for residency education has come under increased review. Step 1 scores will be pass/fail starting in 2022. There has been controversy about grade inflation or the lack of grades altogether at many medical schools. Faculty letters of recommendation have been criticized that they often exhibit implicit bias against women and under-represented minorities or simply are too vague or generic to be useful. Given this difficult landscape, the personal statement carries increased importance to highlight our applicants’ unique motivations and interests. We propose a model that residency and fellowship applicants may employ to craft their personal statements.

## Editorial

From personal statement to mission statement

In recent years, the application process for residency education has come under increased review. Step 1 scores will be pass/fail starting in 2022, increasing the challenge for program directors to critically distinguish between talented applicants [1]. There has been controversy about grade inflation or the lack of grades altogether at many medical schools [2,3,4]. Faculty letters of recommendation have been criticized that they often exhibit implicit bias against women and under-represented minorities [5,6] or simply are too vague or generic to be useful [7,8]. Given this difficult landscape, the personal statement carries increased importance to highlight our applicants’ unique motivations and interests. We propose a model that residency and fellowship applicants may employ to craft their personal statements.

We believe the personal statement is a professional statement. It is your mission statement for why you seek further training. The application is not a prelude to a job interview; it is a request to be considered for further training. Simply put, if you cannot detail why you are applying for a particular residency or fellowship, then do not apply. Your mission statement gives a direction to your planned career. It is the why or the purpose to your direction. Program directors want to see evidence of premeditation, and that you have not arrived by chance or pushed by others. Do not fear any requirement to follow through with specific plans as detailed in your personal statement. All program directors are fully aware that you are likely to change your direction several times during the course of your training. 

While we do not believe there should be a standard form for the personal statement, we do offer general principles, born from decades of experience in reviewing applications for competitive residency programs in internal medicine, medicine-pediatrics, and specialty fellowships. We believe there are recurrent traps that snag bright and well-intentioned applicants:

*1. Be careful of the case vignette: * In our straw poll of last years’ applications, approximately two-thirds included a patient vignette. Much like a TV sitcom, there is a predictable pace with a satisfying end. A troubled patient with a difficult diagnosis is isolated from the healthcare team and has a profound impact on your professional trajectory. The experience inspires you to pursue medicine. 

Our own opinion is to do away with this trope altogether. Maybe this singular experience did move you, but medical education is complex and nuanced. And while this interaction should not be minimized, recounting it does not accomplish the goal of the personal statement. The personal statement is not the proper venue to teach clinical medicine, so even a unique patient presentation detracts from the importance of the central subject, you. As a thoughtful and mature developing professional, the epiphany from a single patient encounter is a worrisome signal of short-sighted thinking when offered as evidence of career selection.

*2. Avoid explaining the program director’s specialty to the specialty program director:* A program director in nephrology knows all about nephrology. They live it. Details about the field in your personal statement does not further your intention of helping clarify your mission statement or make your application stand out. There is an assumption that you enjoy the material, or you would not be applying. Resist the urge to glorify the subject matter to those already convinced.

*3. No need to rehash your Curriculum Vitae (CV): * In the Electronic Residency Application Service (ERAS®) application, the CV is a clean, concise summary of your experience. There is no need to give a detailed description in your personal statement, as its purpose is not to be a narrative form of your CV. As you describe your mission statement, there will of course be elements of your story that appear in both places. Highlight these special accomplishments or milestones in terms of how they shaped your thinking and moved you towards your goal.

*4. Minimize your family’s medical history: * Wrapping your commitment to a life-long profession around the death of a loved one or care of a family member implies a poorly thought out emotionally-tainted decision. Certainly, personal experiences influence your decision, but program directors seek clarity that you have not stumbled your way into your decision to apply by happenstance. You are a professional, and this important decision should never be hurried or rash.

*5. Conclude with a powerful summary: * Never summarize that you are searching for “a supportive teaching program that provides a range of pathology and excellent patient care.” Are there programs that advertise unsupportive faculty with few, homogenous patients, with a narrow range of diseases that are poorly attended? You have done your homework. You would not apply if you think the program lacked essential elements to training. Conclude instead with a powerful and enthusiastic statement that conveys your excitement to begin the work you have outlined.

*6. Be grammatically correct:* It goes without saying (but we will say it anyway), that the personal statement must be grammatically correct, with proper spelling and syntax. Simple errors imply disinterest and inattention. In the era of spell-check and grammar software, take the extra time to ensure there are no grammatical distractions from your message. Have trusted colleagues read and re-read your personal statement. 

The personal statement should clarify why you want to do what you want to do. Examples might include (and have) “I want to pursue pulmonary rehabilitation as it is overlooked as a mainstay of therapy.” Or “Basic neurosurgical techniques are absent in 70% of the hospitals in the United States. I want to work in a small community and help them to gain these advantages.” For a hepatology applicant, “I find great satisfaction in working with marginalized patients typically ignored and stigmatized. Hepatology permits me to help patients with alcohol abuse disorder and work towards preventing end-stage liver disease.” For Internal Medicine, “This is the field that best grapples with the epidemic of self-imposed disease. I want to affect a population with my work.” 

 Each of these statements, ideally, is informed by your CV. Highlighting salient aspects of work you have done strengthens the “why” of your application. The purposeful applicant emerges from the pack as grounded, oriented, displaying evidence of clarity and purpose. Convey more than your enjoyment of the field. In more competitive programs, of course, scholarship that fits with the mission statement enhances the application greatly.

What do you want to talk about at your interview? The personal statement serves as the steppingstone for a high-stakes conversation. Typically, there are aspects of your life that do not pop on your CV that you amplify in the personal statement. What makes you interesting? What will you add to the program’s community? 

The application process is challenging for trainees and program directors alike. The personal statement is the only step in the ERAS application completely under your control. It can make the difference between getting lost in the pile of applications and standing out amongst a competitive group of deserving colleagues. Equally, it is a time to take a look at your professional direction and serve as a milestone for the next leg of your training.
